# Systematic review of the application of virtual reality to improve balance, gait and motor function in patients with Parkinson’s disease

**DOI:** 10.1097/MD.0000000000029212

**Published:** 2022-08-05

**Authors:** Muhammad Kashif, Ashfaq Ahmad, Muhammad Ali Mohseni Bandpei, Maryam Farooq, Humaira Iram, Rida e Fatima

**Affiliations:** a University Institute of Physical Therapy, Faculty of Allied Health Sciences, University of Lahore, Lahore, Pakistan; b Riphah College of Rehabilitation and Allied Health Sciences, Riphah International University, Faisalabad Campus, Faisalabad, Pakistan; c Pediatric Neurorehabilitation Research Center, University of Social Welfare and Rehabilitation Sciences, Tehran, Iran.

**Keywords:** balance, gait, motor function, Parkinson’s disease, physical therapy, virtual reality

## Abstract

**Background:**

Virtual reality (VR) is an advanced technique used in physical rehabilitation of neurological disorders, however the effects of VR on balance, gait, and motor function in people with Parkinson’s (PD) are still debated. Therefore, the systematic review aimed to determine the role of VR on motor function, balance and gait in PD patients.

**Methods:**

A comprehensive search to identify similar randomised controlled trials was conducted targeting 5 databases including Web of Science, PubMed, CINHAL, Cochrane Library, and Physiotherapy Evidence Database. A total of 25 studies were found eligible for this systematic review, and the methodological assessment of the quality rating of the studies was accomplished using the physiotherapy evidence database scale by 2 authors.

**Results:**

Out of the 25 included studies, 14 studies reported on balance as the primary outcome, 9 studies were conducted to assess motor function, and 12 assessed gait as the primary outcome. Most studies used the Unified Parkinson disease rating scale UPDRS (part-III) for evaluating motor function and the Berg Balance Scale as primary outcome measure for assessing balance. A total of 24 trials were conducted in clinical settings, and only 1 study was home-based VR trainings. Out of 9 studies on motor function, 6 reported equal improvement of motor function as compared to other groups. In addition, VR groups also revealed superior results in improving static balance among patient with PD.

**Conclusion:**

This systemic review found that the use of VR resulted in substantial improvements in balance, gait, and motor skills in patients with PD when compared to traditional physical therapy exercises or in combination with treatments other than physical therapy. Moreover, VR can be used as a supportive method for physical rehabilitation in patients of PD. However, the majority of published studies were of fair and good quality, suggesting a demand for high quality research in this area.

## 1. Introduction

As a neurodegenerative and progressive disease, Parkinson’s disease (PD) is multifactorial in nature resulting in deterioration of dopamine-secreting neurons present in the substantia nigra and subsequently leading to the accumulation of Lewy bodies within brain.^[1]^ In some cases of PD, motor symptoms are accompanied by non-motor symptoms, such as sleep disturbance, psychological issues, and constipation.^[2]^ Among all motor symptoms, bradykinesia, rigidity, postural dysfunction, and resting tremor are hallmarks of PD.^[3]^ General population prevalence of PD has been reported to be 0.3% while for the population over 60 years of age it is estimated to be 1% to 2%.^[4]^ Several studies have found higher prevalence and incidence in men as compared to women. Rare cases of Parkinson disease have been found before 50 years of age.^[5]^

PD is managed by a multidisciplinary approach that includes pharmacological and nonpharmacological therapies. Treatment approaches that are currently used for PD are medications, Physical therapy (PT), psychological therapy, nursing care, and surgery.^[6]^ In addition, balance impairment can be improved by deep brain electrical stimulation and dopaminergic stimulation. However, these treatments are expensive and have many contraindications.^[7,8]^ Moreover, improvements as a result of these approaches are not permanent.^[9]^ Complications such as hematomas, paralysis, dislocation, fracture, infection has been reported as a result of surgical procedure and hardware. These complications are not life threatening but main disadvantage of this treatment is expense.^[10]^

No definitive cure is available for PD so symptomatic management is an option commonly used for Parkinsonism.^[7,8]^ Medications have many adverse effects that is, dyskinesia, fluctuations in motor activity, nausea, psychosis and behavioral disorders, when using for symptomatic management of PD.^[11]^ Besides symptomatic management, neurosurgical approaches such as brain stimulation are also used for balance and posture disabilities although drug therapy and neurosurgical approaches are used but still patients feel difficulty in regaining motor activities and independent quality of life.^[11]^

Physical rehabilitation aims to restore functional independence, community participation and prevent secondary complications.^[12]^ The goal of PT in PD is to address posture, gait, balance and physical function of upper and lower extremities.^[13]^ PT approaches used in rehabilitation of PD are balance training, strengthening exercises, stretching exercises, co-ordination exercises, treadmill training.^[14]^ The drawbacks of these PT interventions are loss of follow-up, financial burden and safety of patients.^[15]^

Virtual reality (VR) involves the usage of innovative technologies, including computer interfaces and variant multimedia options for the production of virtual/ simulated environment to provide users with the real-life feel (for the objects and events).^[16]^ VR is characterized by immersion, imagination, and interaction.^[17,18]^ The navigation quality, being another important factor related to VR, is considered an important pre-requisite for enhancing the quality of virtual environment (VE).^[19]^ The main benefits of using VR include cost effectiveness in comparison with other treatment approaches, promotion of active participation, and provision of motivation, and feasibility for home-based use.^[20]^

By using hardware such as a data glove, participants can interact with virtual environments and feel as if they are immersed in the virtual world. Users can gain feedback through different feedback devices and attain interactive feelings and experiences. Different games used in virtual environments are based on the human imagination and cognitive ability. These games are designed to develop interest and motivation.^[21]^ Many studies in the past have shown that VR improves gait parameters in people with CNS disorders.^[22]^ One review stated that there is no difference between home-based VR and conventional training.^[23]^ VR-based exercise provides visual and audio feedback, while conventional balance training provides verbal feedback, and both designs have similar effects on muscle strength and functional performance.^[23]^ The use of information and communication technologies for the sake of delivering rehabilitation to the underserved persons using the electronic means is known as tele rehabilitation (TR). Through TR, the rehabilitation is extended beyond the hospital environment. This also helps in detection of new limitations and for the evaluation of treatment efficacy of the intervention being used in context of activities of daily living, making it another preference for patients with neurological manifestations.^[24]^

Some systematic reviews in the past have noted the effects of VR among individuals with PD, but the conclusions have been conflicting views. Additionally, in current era, research has explored the advantages and disadvantages of both VR and therapies used on routine basis in the management of PD.^[25]^ In addition, VR is being used along with other innovative technologies such as motor imagery as shown in a recent report by Kashif et al.^[26]^ The aim of this systematic review (SR) was therefore to identify the studies that show the effects of VR with or without routine PT on enhancing balance system, improving gait parameters and motor function among patients with PD and also to accomplish the critical assessment and evaluation of the quality of the included studies.

## 2. Methods

A comprehensive search was carried out on 5 online research databases, including physiotherapy evidence database (PEDro), PubMed, Web of Science, Cochrane Library, and CINHAL, using the keywords “Parkinson disease” OR “Parkinsonism” OR “Parkinsonian” AND “virtual reality” OR “Nintendo Wii” OR “Wii Fit” OR “balance board games” OR “Kinect Adventures” AND “motor function” OR “motor skills” OR “bradykinesia” OR “tremor” OR “gait” OR “postural instability” AND “physical therapy” OR “Conventional physical therapy” OR “physiotherapy” OR “physical rehabilitation.”

### 2.1. Research question and study selection

This systematic review was based on the following question: What effect does VR have on balance, gait, and motor skills in PD patients? This question was developed on the basis of population, intervention, comparison, outcome measures, and study design (PICOS) principle.

**Figure 1.**
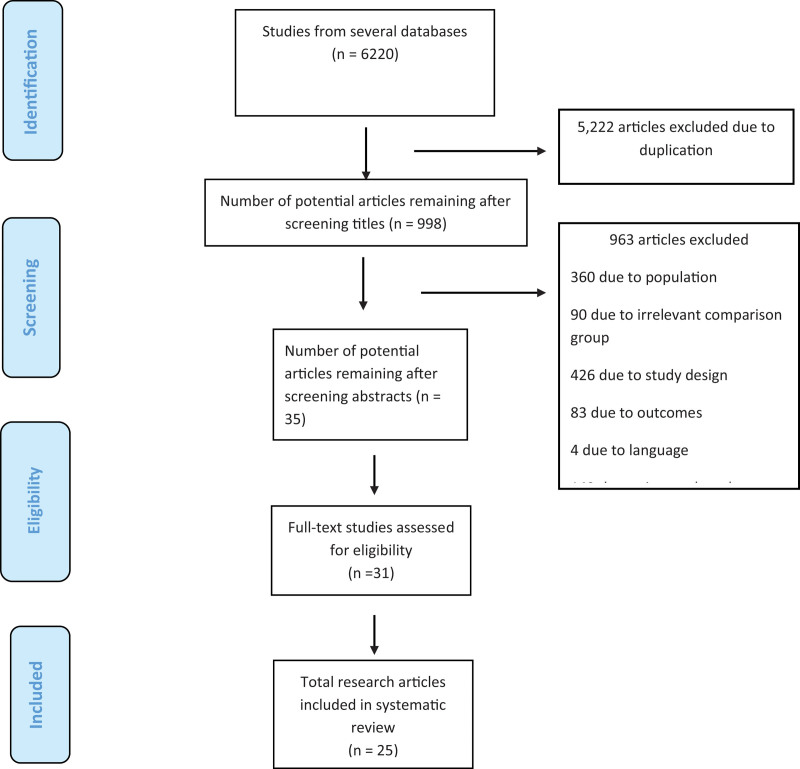
PRISMA Flow diagram for selection of studies.

Previous studies in which the individuals diagnosed with PD were enrolled, where VR as an intervention was compared with or without routine PT and studies with results concerning aspects including balance, gait and motor skills were included in the SR and studies that reported patients with cognitive impairment, hearing impairment, or overt visual impairment were excluded. Only randomized clinical trials and studies in English were included in this systematic review. (Fig. 1).

### 2.2. Data extraction and quality appraisal

Two reviewers used the established strategy from 2002 to 2020 to locate studies for the current systemic review. The data extracted included the age and gender of the participants, sample size, intervention for experimental and control groups, Hoehn-Yahr Scale for measuring Parkinson’s symptoms, dosage used, outcome measurements, and results. The methodological quality assessment of each RCT study included in this review was carried out through use of PEDro scale, which is widely used to qualitatively assess the studies in the physiotherapy literature.^[27,28]^ This instrument is suitable for appraising the methodological quality and completeness of the statistical reporting in SRs for assessing the effects of physiotherapeutic interventions.^[29]^ Therefore, each study was rated using the 11-item PEDro scale. The total PEDro ranged from 0 to 10 and the range for low quality was 0 to 3, for fair quality it was 4 to 5, for good quality it was 6 to 8, and for excellent quality it was 9 to 10.^[30]^

## 3. Results

### 3.1. Study selection and methodological quality assessment (risk of bias)

After searching for the keywords, 6220 articles were retrieved. After screening the titles, 998 studies remained. The number of studies left after screening the abstracts was 31 and finally 25 full-text studies were included in this SR, with 864 participants with PD participating. Random allocation was reported in 24 articles.^[6,23,31–52]^

As per the PEDro appraisal, out of the 25 included studies, Only 1 study^[43]^ scored 3 and considered as poor. Nine studies ^[35,36,38,44,45,49,51,53,54]^ scored 4 to 5 and considered as fair. Fifteen studies ^[23,31–34,37,39,40,42,46–48,50,52,55]^ scored 6 to 8 on Pedro scale and considered as good (Table 1). Data regarding concealed allocation were reported in 8 studies.^[31,32,37–39,49,50,52]^. A total of 16 studies reported blinding of assessors.^[2,23,32–34,36,37,40,42,46,48,50,52,53,55,56]^ Information regarding dropout rates was provided in 7 studies..^[7,23,32,34,42,48,54]^

**Table 1:** Quality assessment of included studies.

Study	Randomly allocation	Concealed allocation	Baseline comparability	Participant blinding	Therapist blinding	Assessor blinding	<15% dropouts	Intention to treat analysis	Between-group difference reported	Point estimate and variability reported	Eligibility criteria	Total score
Hao Feng et al 2019	Y	N	Y	N	N	Y	Y	Y	Y	Y	Y	7
Pompeu et al 2012	Y	N	Y	N	N	Y	N	N	Y	Y	N	5
Shih et al. 2016	Y	Y	Y	N	N	N	Y	N	Y	Y	Y	6
Yang et al 2015	Y	N	Y	N	N	Y	Y	Y	Y	Y	Y	7
van den Heuvel et al 2014	Y	Y	Y	N	N	Y	Y	Y	Y	Y	Y	8
Shen et al 2014	Y	N	Y	N	N	Y	Y	Y	Y	Y	Y	7
Santos et al	Y	N	Y	N	N	Y	Y	Y	Y	Y	Y	7
Robles et al 2016	Y	N	Y	N	N	N	Y	N	Y	N	Y	4
Pazzaglia et al 2019	Y	N	Y	N	N	Y	N	N	Y	Y	N	5
Melo et al 2018	Y	Y	Y	N	N	Y	Y	N	Y	Y	N	7
Ma et al 2011	Y	Y	Y	N	N	N	N	N	Y	Y	N	5
Liao et al 2015	Y	Y	Y	N	N	Y	N	Y	Y	Y	Y	7
Liao et al 2014	Y	Y	Y	N	N	Y	Y	N	Y	Y	Y	7
Gandolfi et al 2017	Y	N	Y	N	N	Y	Y	N	Y	Y	Y	6
Ferraz et al 2018	Y	Y	Y	N	N	Y	Y	N	Y	Y	Y	7
Cikajlo et al 2019	Y	N	N	N	N	N	Y	N	Y	Y	Y	4
Yuan et al ^[43]^	Y	N	N	N	N	N	N	N	Y	Y	Y	3
Su et al ^[44]^	Y	N	Y	N	N	N	Y	N	Y	Y	Y	5
Barros et al 2014	Y	N	Y	N	N	N	Y	N	Y	Y	Y	5
Mirelman et al 2014	N	N	Y	N	N	N	Y	Y	Y	Y	Y	5
Ilaria Carpinella et al 2016	Y	N	Y	N	N	Y	Y	N	Y	Y	Y	6
Moon et al 2020	Y	N	Y	Y	Y	N	Y	N	Y	Y	Y	7
Lee et al 2015	Y	N	Y	N	N	N	Y	N	Y	Y	Y	5
Tollar et al 2018	Y	N	Y	N	N	Y	Y	N	Y	Y	Y	6
Ribas et al 2017	Y	Y	Y	N	N	Y	Y	N	Y	Y	Y	7

### 3.2. Study designs and population characteristics

Of the 25 studies included in this SR, all studies were published from 2000 to 2020. The interventional groups present in the included studies reported the use of VR-based protocols in the form of balance training,^[6,23]^ sports games,^[31]^ visual feedback training,^[32]^ treadmill training with nonimmersive VR,^[33]^ gait training,^[33]^ functional training groups,^[41]^ 3D Oculus Rift CV1 pick-and-place tasks in a virtual world,^[51]^ treadmill training with nonimmersive VR, interactive video-game-based exercise,^[43]^ projection-based VR system and balance board use,^[44]^ virtual exercises such as aerobic and balance exercises,^[45]^ VR and treadmill training,^[57]^ game pad system use,^[46]^ balance training using Wii Fit (BTWF) and traditional occupational therapy,^[47]^ VR dance exercise with neurodevelopment training and functional electrical stimulation,^[49]^ and exergaming.^[50,52]^

### 3.3. Sample characteristics

Eight hundred sixty four PD participants were included in this SR. Majority of studies included participants from both genders except 1 study which included only male patients with PD.^[34]^ The total male population was 529 in all included studies. The maximum number of male participants was 51,^[40]^ and the minimum number was 10.^[49]^ The total population of female participants in all studies was 335. The maximum number of participants was 25,^[41]^ and the minimum number was 3.^[35]^ (Table 2)

**Table 2:** Data extraction of the included studies.

Study	Age	Gender	Sample size	Experimental group	Control group	Hoehn-Yahr scale	Dosage	Outcome measures	Conclusion
Pompeu et al 2012	Mean 67.4	M/F: 17/15	32	Wii based training	Traditional training (Balance Exercises)	1–2	I hour/Day, 2 days/week, Total 7 wks	BBS,UPDRS, MCA	The Wii based training and traditional training exercises do not have a significant difference in improving balance in patients with PD
Shih et al 2016	EG: Mean: 67.5 CG: Mean 68.8	M/F: 16/4	20	Balance-based exergaming game	Conventional balance training	1–3	2 sessions every week, for 8 wks	LOS, OLS, BBS, TUG	Compared to conventional training, exergaming creates significant improvements in postural stability.
Yang et al 2015	EG: Mean:72.5 CG: Mean:75.4	M/F:14/9	23	Customized VR balance training system	Static posture stability and weight shifting in dynamic posture	3	50 minutes/ session (6 wks in total)	UPDRS-III, BBS, TUG test	VR based balance training was equally effective as training carried out in home environment for improvising balance function, ability to walk, and quality of life.
van den Heuvel et al 2014	EG: mean:66.3 CG: Mean: 68.8	M/F: 20/13	33	VFT	Conventional training	02–03	Ten group treatment session of 60 min duration, for 5 wks	FRT, SLS, 10 MWT, UPDRS, BBS, TUG test	VFT appeared to be practicable and harmless approach. However, no significant difference was found in any outcome measure.
Shen et al 2014	EG:Mean 63.3 CG: Mean 65.3	M/F: 25/20	45	balance and gait training with VFT	lower-limb strength training	2.5	Training for 12 wks	ABC scale,**LoS**, **SLS**, and spatiotemporal gait characteristics	Positive effects of augmented feedback training. P0 <0 0.05 in ABC score, Gait velocity, stride length
Santos et al (2019)	NW group: mean 61.7CE group: mean: 64.5NW+CE:mean: 66.6	M	45	Nintendo Wii(NW) game	2 control groups1. Nintendo wii + conventional training2. conventional training group	01–03	50 min, twice a week	BBS, DGI, TUG, PDQ-39	In rehabilitating PD patients, the Nintendo Wii plus conventional exercises was statistically as effective as any intervention alone; however, the combination was more effective.
Robles et al 2016	EG: mean 68.75 CG: mean 64.2	M/F: 12/3	15	Full-amplitude repetitive finger-tapping (FT)	VR captured patients’ self-movements		4-wks of training	Finger-tapping test, Cortico-spinal excitability	The intervention group exhibited greater movement amplitude after treatment, therefore, indicating that VR can assist in improving motor function within the context of movement imitation therapy.
Pazzaglia et al 2019	Age (years)0 =0 CG: 70 EG:72	M/F: 35/16	51	VR training for consecutive 6 weeks with 400 min session 3 Times/week	Conventional rehab program for consecutive 6 weeks with 400 min session 3 Times/week		40-min session three times per week	BBS, DGI, DASH, Short SF-36	A VR protocol was superior to a conventional rehabilitation program for improving balance.
Melo et al 2018	Control: 65.580 ±0 13.04, Treadmill group: 610 ±0 10.72, VR group: 60.250 ±0 9.28	M/F: 28/9	37	treadmill group, VR group	Conventional training group	01–03	20-min training sessions three times a week for 4 wks	6MWT, Inertial Measurement Unit (IMU)	Patients with PD in both groups did not experience any difference in treatment outcomes.
Ma et al 2011	EG: mean 64.77 CG: mean 68.13	M/F: 18/15	33	VR training	Placebo Training	02-03	60 trials	Movement time, peak velocity and percentage of movement time for acceleration phase	A brief VR training program improved the speed of movement of discrete target tasks while reaching for real moving objects, but therapy had a minimal effect on performance.
Liao et al 2015	CG:64.60 ±0 8.6 TE:65.10 ±0 6.7 VRWii:67.30 ±0 7.1	M/F: 17/19	36	VRWII group Traditional exercises group	Education group	01–03r	2 sessions per week over a 6-wk period	Level walking performance, FGA, Hand held dynamometer	VRWii is an exercise program that has shown effectiveness for patients with PD in improving their ability to walk and muscular strength..
Liao et al 2014	CG:64.60 ±0 8.6 TE:65.10 ±0 6.7 VRWii:67.30 ±0 7.1	M/F: 17/19	36	VR WII group Traditional exercises group	Education group	‘1–3	2 sessions per week) over a 6-wk period	LOS,PDQ-9), (FES-), and (TUG)	Compared to traditional exercises, VR Wii training significantly increased dynamic balance in patients with PD, which assisted the use of VR Wii to improve balance.
Gandolfi et al 2017	Tele wii group:67.45control group:69.84	M/F:51/25	76	Home based Nintendo Wii fit training	Clinical based exercises targeting postural stability of the patients	2.5–3	Total of 21 sessions, 50 min duration/session, 7 successive weeks	BBS,ABC10-MWT, DGI	VR training at home significantly improved balance and postural instability compared to clinic based balance exercises in patients with PD.
Ferraz et al 2018	60 years of age	M/F: 37/25	62	Functional training groupBicycle exercise group	Kinect adventure group	2–3	three 50-min sessions per week for 8 wks	6MWT, 10MWT,SRT,PDQ-39	This study found that exergaming can improve the ability to walk in older people with PD. However, they have the same effect compared to bicycle training and functional training.
Hao Feng et al 2019	EG:67.47CG:66.93	M/F:17/13	28	VR training	conventional physical therapy	2.5–04	45 each session, 5 days/week, total duration 12 wks	BBS, TUGT, UPDRS-III, and FGA	The study results depicted that balance and gait were improved significantly in VR training group.
Cikajlo et al 2019	2D group: 71.30 ±0 8.4, 3D group: 67.60 ±0 7.6 years old	M/F	20	3D Oculus Rift CV1, Pick and place task in virtually created environment	Pick and place task in virtual environment	02–03	10-session 3 wks	Leap motion controller, (BBT), and IMI	3D immersive VR technology enhances the interest / pleasure worth resulting in robust and efficient physical functioning, and is a superior enhancement over 2D technology.
Yuan et al 2020	EG: mean 67.8, CG: mean 66.5	M/F: 11/13	24	Interactive video-game–based exercise (IVGB)	No interactive video-game–based Exercise in control phase	01–03	6–12 wks	BBS, SF-36, MFES, MSL	IVGB exercise training can improvise physical function as an alternate therapy regime among mild to moderate presentation of PD.
Su et al 2014	EG: 64.76 CG: 64.71	M/F:26/16	42	projection-based VR system and balance board	projection-based VR system and balance board	02–03	15 test trials for each difficulty level of selected game, with a 5-min break	Wii balance board kinematic variables such as peak velocity, end of movement	Varying the speed of moving objects in VR system has an impact on speed of arm and COP among PD patients
Barros et al 2014	45–85 y old	M/F	15	Virtual exercises such as aerobic and balance exercises		02–04	14 session in total, twice per week (each session lasting for 40 min)	(UPDRS III), SE-ALD, FIM, and biomechanical analysis of gait	Nintendo Wii Fit Plus platform training was effective and efficient in a short time period for improving gait.
Mirelman et al 2014	Mean: 67.1	M/F: 14/6	20	VR and treadmill training	VR without treadmill training	02–03	6 wks (three sessions per wk)	Accelerometer, UPDRS- III, (PDQ-39), MCA	Treadmill training + VR significantly improve physical performance and gait.
Ilaria Carpinella et al 2016	Average 70 y	M/F	42	Game pad system	Structured physiotherapy without feedback	02–04	20 sessions of 45 min each, 3 times a wk	BBS, 10 meter walk test, Tele-healthcare Satisfaction questionnaire	Statistically significant between-group differences in BBS p0 =0 0.047
Moon et al 2020	EG: mean 63.38, CG: mean 62.14	M/F: 10/5	15	balance training using Wii Fit (BTWF)+ traditional occupational therapy	traditional occupational therapy	02–03	3 times/ week, in total for 8 weeks and 30 minutes of Wii fit training in each single session	BBS, TUG, MBI	The Wii Fit training resulted in significant changes in BBS score in comparison to the control group.
Lee et al 2015	EG: mean 68.4, CG: mean 70.1	M/F: 10/10	20	VR dance exercise plus neurodevelopment training plus functional electrical stimulation	neurodevelopment training plus functional electrical stimulation		6 wks	BBS, the MBI Index, and the BDI	VR dance exercises have a positive effect on balance, the ADLs and the depression status of Parkinson’s patients with P value0 <0 0.05.
Tollar et al 2018	EXE:70.00 ±0 4.69 CYC:70.60 ±0 4.10 CON: 67.50 ±0 4.28	M/F36/38	74	Exergaming	Stationary cycling (CYC) andWaitlist	2–3	60 min/d 5 d/w5 w	PDQ-39BBS	VR group improved significantly in BBS scores in comparison to the control group.
Ribas et al 2017	610 ±0 9.11	M/F8/12	20	ExergamingWii fit games	Conventional exercise	1–3	30 m/d2 d/w12 wks	BBSPDQ-39	Short-term, significant improvements in BBS.

### 3.4. VR with routine physical therapy

Out of the 25 studies, 12 were conducted to determine the effects of VR in comparison with routine PT. The types of physical therapies used in these studies were conventional balance training,^[31,50]^ dynamic weight shifting,^[23]^ standing balance exercises,^[23]^ stepping exercises,^[32]^ dual task exercises,^[32]^ strengthening exercises,^[33]^ motor coordination exercises,^[36]^ stretching exercises,^[39]^ fall prevention exercises and walking training,^[39]^ structured physiotherapy without feedback,^[46]^ and clinical-based exercise.^[40]^

The types of VR applications used in these studies were VR augmented visual feedback training balance,^[32]^ board exergaming involved reaching and obstacles avoiding tasks,^[32]^ SMART EquiTest Balance Master,^[33]^ Nintendo Wii,^[34]^ Nirvana optoelectric device,^[36]^ exergaming,^[50]^ Gamepad system,^[46]^ home-based Nintendo Wii Fit system,^[40]^ balance-based exergaming,^[31]^ VR Wii,^[39]^ and a custom-made VR system.^[23]^

### 3.5. VR without routine physical therapy

Out of the 25 studies, 13 compared VR with other treatment approaches. These other treatment approaches were imitation therapy with motor practice,^[35]^ Sensory Integration Balance Training (SIBT),^[40]^ traditional occupational therapy,^[47]^ VR without treadmill training,^[57]^ and bicycle exercise neurodevelopment training with functional electrical stimulation.^[49]^ The types of VR used in these studies were Tele Wii^[40]^ and Xbox Kinect.^[41]^

### 3.6. Method of intervention

In all studies, pre-post training was incorporated. Interventions in most of the studies lasted 6 weeks. The training sessions lasted from 30 to 60 minutes. A total of 7 studies^[36,38,42,44,45,49,51]^ did not report a follow-up, and 6 studies^[6,23,33,34,40,41]^ reported the retention of the effects.

### 3.7. Home-based or clinical-based

Out of the 25 studies, 24 reported clinical-based training, and 1 study^[23]^ was home-based. For those patients who were unable to come in clinical setup from remote areas to participate in the clinical trials, technology-based tools were provided to them with home-based assessments. This reduced the duration and frequency of visits and also provided motivation to depressed patients. Such an approach is also cost effective for both patients and clinicians.^[28]^

### 3.8. Outcomes

#### 3.8.1. Balance.

Out of the 25 studies, 14 assessed balance as an outcome.^[23,31–34,36,40,42,46,47,50,52,57,58]^ In 11 studies, balance was assessed as a primary outcome, and in 1 study,^[57]^ balance was assessed as a secondary outcome. Out of these 12 studies, 4 studies ^[31–33,43]^ assessed both static and dynamic balance. All studies reported gains in Berg Balanxe Scale (BBS) and Time up and go test (TUGT) scores; some studies concluded that VR is an effective tool for the intervention^[59]^ and a good alternative to exercises alone.^[60]^ Studies with follow-ups ^[6,23,33,34,40,41]^ suggested that the effects of VR can be retained after discontinuation of treatment. The home-based VR system provided gains in balance.^[23]^ Four studies ^[23,32,48,53,58]^ reported similar effects of VR and conventional PT in improving balance. One study^[54]^ reported that a combination of VR and treadmill training revealed positive findings in the reduction of falls as compared to treadmill alone.

### 3.9. Outcome measures for balance

In all 14 studies, outcome measures used for balance assessment were the BBS, TUG, Functional Reach Test (FRT), Limits of Stability (LOS), One-Legged Stance Test (OLS), motor section of the Unified Parkinson’s Disease Rating Scale (UPDRS), and the Activities-Specific Balance Confidence Scale. The majority of the studies used the BBS as the primary outcome measure for functional balance.

### 3.10. Gait

Out of the 25 studies, 12 assessed gait to determine the outcome of the study.^[23,32,34,36,37,42,45,46,48,53,55,57]^ In 5 studies,^[37,45,46,48,57]^ gait was assessed as a primary outcome, and in 7 studies,^[23,32,34,36,42,53,55]^ gait was assessed as a secondary outcome. Out of these 12 studies, 7 studies ^[34,36,42,46,48,55,57]^ reported significant differences in gait variables as compared to the control group. One study^[37]^ reported the same effects of VR and treadmill training on gait parameters. One study^[53]^ was conducted on the improvement of stationary gait through Wii Fit games. Another study^[55]^ used three types of intervention groups: functional training, bicycle exercise, and exergaming. After the intervention, only the exergaming group revealed substantial improvements on the 10 meter walk test (MWT). The assessment of gait was performed on including gait speed,^[46]^ crossing stride length, crossing stride velocity, vertical toe obstacle clearance,^[48]^ distance, and symmetry index.^[37]^

### 3.11. Outcome measures for gait assessment

Outcome measures used for gait assessment were a 10-metre walk test,^[32,46,55]^ functional gait assessment,^[42]^ 6-minute walk test,^[55]^ Liberty system to assess obstacle crossing performance,^[48]^ inertial measurement unit,^[37]^ a GaitRite Mat and accelerometer,^[57]^ Dynamic Gait Index,^[23,34,36]^ and the Freezing of Gait Questionnaire.^[46]^

### 3.12. Motor function

Out of the 25 studies, 9 studies ^[23,32,35,38,42,44,45,51,57]^ were conducted to assess motor function. Of 9 studies, only 3 studies ^[23,32,42]^ assessed motor function as a secondary outcome. Six studies ^[23,32,35,38,42,45]^ conducted on improvement of motor function reported no significant difference between groups, and 3 studies ^[36,51,57]^ reported significant differences in motor function-related outcome measures. One study^[57]^ reported 7 points of significant difference only in the domain of mobility on the UPDRS-III. Another study was done by Su et al^[43]^ to evaluates the effects of moving target speed on arm movement, who reported that fast balls produced lower success rates and powerful arm movements compared to slow balls.^[45]^ Ma and fellows in 2011 carried a research to compare the results of using the stationary balls and with balls moving at different speeds. They found that the movement was faster and more powerful in the VR group. However, no difference was found in the success rate and kinematics of movement.^[38]^

### 3.13. Outcome measures for motor function assessment

Outcome measures for assessing the motor function included the UPDRS-III,^[23,32,42,45,57]^ Wii Balance Board and Patriot Motion Sensor,^[45]^ finger tapping test, Biometrics Data LINKS system, corticospinal excitability,^[57]^ electromagnetic motion tracking system,^[38]^ and motion trajectory analysis.^[51]^

### 3.14. Other outcomes assessed along with balance, gait, and motor function

After data extraction from the 25 studies, we found secondary outcomes such as quality of life,^[23,34,43,45,57]^ fall risk,^[43]^ health status and level of activity/participation,^[32]^ physical function and symptoms in upper limb,^[36]^ cognitive function,^[53,57]^ muscle strength,^[39]^ emotional state,^[55]^ and level of satisfaction^[46]^ that were assessed along with balance, gait, and motor function. Significant differences were found in fall risk,^[43]^ physical function and symptoms in upper limb,^[36]^ mental domain of quality of life,^[36]^ activities of daily living, ^[45,47,49]^ and cognitive function.^[57]^

Outcome measures included the Short form (SF)-36,^[43]^ Modified Fall Efficacy Scale,^[43]^ Parkinson Disease Quality of Life Scale (PDQ-39),^[23,34]^ Multidimensional Fatigue Inventory,^[32]^ Disabilities of Arm, Shoulder, and Hand,^[36]^ Functional Independence Measure, Schwab, and England Daily Living Activities Scale^[45]^ Montreal Cognitive Assessment,^[53,57]^ Handheld Dynamometer,^[39]^ Sitting-Rising Test,^[55]^ Geriatric Depression Scale,^[55]^ Tele-Healthcare Satisfaction Questionnaire,^[46]^.

## 4. Discussion

In current era, VR has appeared to be an advance, innovative technology for physical rehabilitation purpose. Because of its unique use in training methods and its function as a personalized rehabilitation tool, it has gained popularity in PD rehabilitation.^[61]^ Therefore, some platforms based on professional technology and VR based rehabilitation systems are under development, and many researchers are working on the application of VR as a neural rehabilitation tool.^[62–64]^. This SR was aimed to assess the effectiveness of VR with or without routine PT treatment in improving balance, gait and motor function among patients with PD.

### 4.1. Balance

A number of neurological disorders, including PD, are associated with balance impairments.^[65]^ The findings of recent review are in line with the previous SRs on Stroke and Alzheimer’s disease,^[66,67]^ proposing that conventional physiotherapy intervention is inferior to VR technology in improving balance and other subject impairments. Several studies trained patients by using somatosensory game software, and a few studies used traditional exercises as a basis for VR technology application. Each training session time duration ranged from 30 to 60 minutes. However, the frequency of the training sessions varied in different studies. Several studies applied training 2 to 3 times in a week, while others carried out training 5 times/week. The treatment was carried out for 4 to 12 weeks period. This heterogeneity of results can be attributed to the fact that the type of VR intervention and traditional PT interventions were different in each study. Presently, no study is available that shows what kind of VR intervention and which treatment intensity have more beneficial effects. According to the Hoehn-Yahr Scale, in different clinical stages, PD patients have varied tremor amplitudes and rhythm patterns that increase muscle tension and levels of indolence as the disease progresses.^[15]^ Another cause of the heterogeneity of our study results is the inclusion of different stages of the Hoehn-Yahr Scale in different studies, which affected the results of the treatment intervention. To eliminate such variability in the results, future studies can be conducted to target different interventions for different disease stages.

Another study conducted to improve balance confidence by augmented feedback training reported the longest carryover effects and sustained improvement for 12 months in rapid and large steps and a narrowing of the base of support.^[33]^ One study reported that a combination of Nintendo Wii and conventional exercise improves gait, mobility, and quality of life,^[34]^ whereas another did not recommend VR in cases of severe cognitive deficits.^[36]^ One study used the comparison of Tele Wii and SIBT and reported superior effects of Tele Wii.^[40]^

In the VR group, balance and gait measures both improved at the 3- and 12-months follow-up periods, but no improvement was reported in the control therapy group. These results are based on a single trial, so further research is required for investigating the long-term effects of VR exercises.^[33]^

### 4.2. Gait

In the majority of the trials, active control intervention was closely linked with conventional physiotherapy programmes. Tomlinson and colleagues conducted a SR and found that conventional physiotherapy chiefly affects gait and balance function.^[68]^ These results are supported by the current review indicating that VR exercises have greater effects on gait and balance function. When comparing gait and balance improvement, VR was found to be more effective for improving step and stride length as gait parameters, while balance function (composite measure) was approached significance in support of VR.

A characteristic feature of PD related to gait is a decrease in step and stride length, while other gait-related manifestations include decreased gait speed, enhanced variations in gait pattern, and increased double-stance time among this population.^[69]^ In PD, the capability to generate a normal gait pattern is not affected; however, the automatic gait control mechanism decreases, and attention strategies are required to improve the automaticity.^[70]^ VR technology may provide more precise and absolute motor feedback, and thus it can greatly improve stride amplitude A study conducted recently concentrated on VR treadmill training, and the results signified enhanced step and stride length as well as gait speed through VR-based gait training.^[71]^ In contrast to other symptoms, postural instability is another disabling symptom of PD^[72]^ that responds weakly to dopaminergic therapy.^[73,74]^ For that reason, postural instability may be greatly improved by PT interventions both with and without VR.

### 4.3. Motor function

The results of our review reported significant differences in motor function-related outcome measures. Numerous studies have shown that patients learn motor skills in virtual environments, and that these are easily implemented in real life.^[17,75]^ VR helps with adjusting limb alignment in games and determining the direction of movement and position through a combination of visual and sensory information.^[51,76]^ Patients in virtual environments perform tasks repeatedly, gain feedback about performance, and enhance motivation, which is critical in patients with PD.^[22]^

Virtual reality improves cognitive and motor skills, such as attention and executive function, by combining them. This makes it easier for people to be more independent.^[40]^ he central nervous system (CNS) generates new pathways through visual and auditory feedback, which is the actual mechanism underlying such learning. Motor function is enhanced by the activation of mirror neurons in the cerebral cortex. Postural instability is another prominent feature of PD that is associated with vestibular dysfunction.^[77]^ VR games can improve vestibular function and stability, and physical rehabilitation and exercise therapy can decrease oxidative stress and increase the release of neurotransmitters.^[58]^ One study reported no benefits of exergaming on bradykinesia and similar effects of exergaming and balance training on functional balance.^[31]^

The current review investigated the role of VR technology in rehabilitating patients with PD. Using the rehabilitation protocol, initially, the motor functioning of patients can be greatly improved by using sports training VR interventions that work on restructuring the central lateral sensory-motor cortex. Brain functions like perceiving, processing, and information integration can also be improved by focusing on enhanced balance and postural control. VR technology can also help patients stay on track with rehabilitation training and be more interested by letting them experience different environments through different sensors and giving them immediate feedback on their audio-visual senses.^[33]^

VR intervention can offer new individualized training therapies according to an individual patient’s disease characteristics, when compared with routine PT. In addition, VR use has other advantages as well; in real time, it can upload training data on the internet and can also endorse patient and health system relationships, as it can synchronize data between various devices, thereby improving the overall rehabilitation effect.

In brief, most of the studies’ quality of evidence was found regarding the use of VR-enhanced exercise as a valuable tool to improve motor function, gait, and balance impairment in PD patients. However, the quality of the studies included was not very high, so further studies should be conducted to produce the quality of evidence. More research is required to evaluate the efficacy of VR technology before its implementation is promoted further. It would also be valuable to determine which type of VR is more useful for motor rehabilitation and other Parkinson’s-related impairments.

### 4.4. Limitations

The present study has some limitations that should be addressed. Limitations reported in studies were small sample size, loss of follow-up, and lack of generalizability. Even though a comprehensive literature search has been conducted, there are chances that few studies are omitted (i.e., grey literature and studies in other languages). Second, in the treatment group, the patients could not be blinded because of the specificity of VR intervention, so this could lead to a change in subjective data when evaluating results. Although a double blind design is best for RCT studies, it is certainly difficult to apply this method in this case. In conclusion, in the majority of studies, financial comparisons were not performed between the control and experimental groups, and only 1 study reported the cost of training through VR intervention. Further studies should conduct cost comparisons of different rehabilitation interventions.

## 5. Conclusion

This SR found that using VR in rehabilitation can bring more significant improvements in balance, gait, and motor skills in patients with PD in comparison to traditional PT exercises or in combination with treatments other than PT. VR should therefore be used as a supportive method of rehabilitation.

## Author contributions

Conceptualization: Muhammad Kashif, Ashfaq Ahmad, Muhammad Ali Mohseni Bandpei.

Data curation: Muhammad Kashif, Ashfaq Ahmad.

Formal analysis: Muhammad Kashif, Humaira Iram. Rida e Fatima.

Investigation: Muhammad Kashif, Maryam Farooq, Humaira Iram.

Methodology: Muhammad Kashif, Maryam Farooq, Ashfaq Ahmad, Rida e Fatima.

Project administration: Ashfaq Ahmad, Muhammad Ali Mohseni Bandpei.

Resources: Muhammad Kashif, Maryam Farooq, Rida e Fatima, Humaira Iram.

Software: Maryam Farooq, Rida e Fatima.

Supervision: Ashfaq Ahmad, Muhammad Ali Mohseni Bandpei.

Validation: Ashfaq Ahmad, Muhammad Ali Mohseni Bandpei.

Visualization: Muhammad Kashif, Ashfaq Ahmad, Muhammad Ali Mohseni Bandpei.

Writing – original draft: Muhammad Kashif. Ashfaq Ahmad, Muhammad Ali Mohseni Bandpei.

Writing – review & editing: Muhammad Kashif, Ashfaq Ahmad, Muhammad Ali Mohseni Bandpei,
